# Predictive Biomarkers of Bacillus Calmette-Guérin Immunotherapy Response in Bladder Cancer: Where Are We Now?

**DOI:** 10.1155/2012/232609

**Published:** 2012-08-07

**Authors:** Luís Lima, Mário Dinis-Ribeiro, Adhemar Longatto-Filho, Lúcio Santos

**Affiliations:** ^1^Experimental Pathology and Therapeutics Group, Portuguese Institute of Oncology, 4200-072 Porto, Portugal; ^2^ICBAS, Abel Salazar Biomedical Sciences Institute, University of Porto, 4099-003 Porto, Portugal; ^3^Nucleo de Investigação em Farmácia, Centro de Estudos em Saúde e Ambiente (CISA), School of Allied Health Sciences, Polytechnic Institute of Oporto, 4400-330 Vila Nova de Gaia, Portugal; ^4^Department of Gastroenterology, Portuguese Institute of Oncology, 4200-072 Porto, Portugal; ^5^CINTESIS/Department of Biostatistics and Medical Informatics, Faculty of Medicine, University of Porto, 4200-319 Porto, Portugal; ^6^Laboratory of Medical Investigation (LIM) 14, Faculty of Medicine, University of São Paulo, 01246903 São Paulo, SP, Brazil; ^7^Life and Health Sciences Research Institute (ICVS), School of Health Sciences, University of Minho, and ICVS/3B’s-PT Government Associate Laboratory, 4710-057 Braga, Portugal; ^8^Molecular Oncology Research Center, Barretos Cancer Hospital, 1331 Barretos, 1478-400 São Paulo, SP, Brazil; ^9^Health Sciences Faculty, University Fernando Pessoa, 4200-150 Porto, Portugal; ^10^Department of Surgical Oncology, Portuguese Institute of Oncology, 4200-072 Porto, Portugal

## Abstract

The most effective therapeutic option for managing nonmuscle invasive bladder cancer (NMIBC), over the last 30 years, consists of intravesical instillations with the attenuated strain Bacillus Calmette-Guérin (the BCG vaccine). This has been performed as an adjuvant therapeutic to transurethral resection of bladder tumour (TURBT) and mostly directed towards patients with high-grade tumours, T1 tumours, and *in situ* carcinomas. However, from 20% to 40% of the patients do not respond and frequently present tumour progression. Since BCG effectiveness is unpredictable, it is important to find consistent biomarkers that can aid either in the prediction of the outcome and/or side effects development. Accordingly, we conducted a systematic critical review to identify the most preeminent predictive molecular markers associated with BCG response. To the best of our knowledge, this is the first review exclusively focusing on predictive biomarkers for BCG treatment outcome. Using a specific query, 1324 abstracts were gathered, then inclusion/exclusion criteria were applied, and finally 87 manuscripts were included. Several molecules, including CD68 and genetic polymorphisms, have been identified as promising surrogate biomarkers. Combinatory analysis of the candidate predictive markers is a crucial step to create a predictive profile of treatment response.

## 1. Introduction

Thirty years have passed, and intravesical instillations with the attenuated strain bacillus Calmette-Guérin (BCG) are still considered the most effective adjuvant treatment for non-muscle invasive bladder cancer (NMIBC). Generally this treatment is performed adjuvant to transurethral resection of bladder tumour (TURBT) in intermediate and especially high-risk NMIBC, such as, patients with high-grade tumours, T1 tumours, carcinoma *in situ *(CIS), multiple tumours, large volume tumours, and high rate of prior recurrence tumours [1].

Recent systematic reviews and meta-analysis have shown that BCG therapy contributes to a significant reduction of recurrence and disease progression for high-risk patients and CIS when compared to TURBT alone or intravesical chemotherapy [2–4]. However, several studies demonstrated that from 20% to 40% of the patients fail to respond to this therapeutic, which may result in tumour progression [5–9]. Other important fact related with BCG treatment is that 90% of patients will experience some sort of side effects (local cystitis symptoms such dysuria, frequency alteration, and occasional haematuria) [10, 11] and, for this reason, an elevated number of patients did not complete the treatment schedule [12, 13] although a significant higher withdrawal rate of patients treated with BCG could not be demonstrated [12–14].

Since the response to BCG is unpredictable, it is important to find a reliable predictive biomarker and/or a marker that could identify elevated risk groups of treatment failure and side effects development. Currently, no markers are available to predict BCG response (neither clinicopathologic, immunological, inflammatory nor genetic markers).

Biomarkers are defined as “a characteristic that is objectively measured and evaluated as an indicator of normal biological process, pathogenic process, or pharmacological responses to a therapeutic intervention.” Predictive biomarkers will foretell how the patient is going to respond to a given therapy. A predictive marker predicts response or resistance to a specific therapy, whereas a prognostic marker, as described above, predicts relapse or progression independently of future treatment effects. Many markers may have both a prognostic and a predictive value [15].

There is some controversial among studies regarding clinical and histopathological predictive factors; therefore, up-to-date none of these markers have demonstrated a reliable predictive role in BCG response, possibly because the NMIBC population candidate for BCG therapy was already selected for its aggressive potential.

Despite intensive research, the exact mechanisms involved in BCG therapy remain elusive. One of the major goals for the next years is the identification of a reliable set of immunological predictive factors, which would allow the identification of responders and nonresponders prior to or at the beginning of immunotherapy. In particular, this may permit the early identification of those patients who suffer the more unpleasant and potentially hazardous side effects associated with BCG therapy, enabling them to be offered alternative treatment [16].

Therefore, the purpose of this systematic review is to conduct a critical analysis of the available literature in order to assess molecular markers (predictive biomarkers) found to be related with BCG treatment recurrence and progression. To the best of our knowledge, this is the first systematic reviews focusing only on molecular predictive biomarkers of BCG treatment outcome.

## 2. Material and Methods

A systematic review was conducted through a MEDLINE database (PubMed) search, in order to retrieve papers linking biomarkers associated with BCG treatment outcome, available online in July 2011, using the following query: ((“Urinary Bladder Neoplasms”[Mesh] OR “bladder cancer”[All Fields] OR “superficial bladder cancer”[All Fields]) AND (“BCG Vaccine”[Mesh] OR “bcg”[All Fields] OR “bcg treatment”[All Fields] OR “BCG immunotherapy”[All Fields] OR “BCG therapy”[All Fields] OR “intravesical therapy”[All Fields] OR “Bacillus Calmette-Guérin”[All Fields])) AND (“Neoplasm Recurrence, Local”[Mesh] OR “recurrence”[All Fields] OR “outcome”[All Fields] OR “treatment failure”[All Fields]).

Through this search 1324 abstracts were gathered and then read. Inclusion/exclusion criteria were created to retrieve only papers focusing molecular markers and BCG immunotherapy response published before 1995. Finally, the reference list of all selected publications and review articles excluded was also checked for additional studies missed on the PubMed search; therefore, two studies were included. Finally, 87 manuscripts were included. Selected studies were then characterized in a structured sheet, the quality assessed, and the pooled data analyzed.

The quality of papers was also independently assessed by two researchers (LL and LS). The quality of the studies was assessed using an eight-item quality assessment scale, based on STROBE Statement [17]. Each item had a score of 1, and the mean quality score of all 87 manuscripts was 5,26/8.

 Predictive factors (biomarkers) found were divided in three major categories, such as “Tumour molecular characteristics” with 34 papers that analysed a total of 40 tumour molecular characteristics (mean quality score was 5,13/8), “Urinary markers” 18 which were evaluated in a total of 21 published papers (mean quality score was 4.62/8), and “Genetic Polymorphisms” with 17 papers published studying 65 genetic polymorphisms in 36 genes (mean quality score was 6.33). The outcomes evaluated were recurrence, recurrence-free survival (RFS), progression, and progression free Survival (PFS).

## 3. Results

 Using the criteria defined in the material and methods section several biomarkers related with BCG treatment have been identified and organized according to their biological nature. This information has been comprehensively summarized in Tables 1, 2, and 3. In particular, Table 1 refers to molecular characteristics evaluated in the tumour prior to treatment, Table 2 refers to urinary markers measured during treatment, and Table 3 compiles information about genetic polymorphism evaluated in the context of BCG treatment response. The most promising biomarkers are presented in more detail the following sections.

### 3.1. Tumour Molecular Characteristics

#### 3.1.1. p53

p53 is a well-known protein involved in cell cycle and apoptosis regulation, its expression was the evaluated in 18 studies, making it the most studied molecular tumour marker. p53 expression showed no correlation with recurrence rate after BCG treatment in none of the studies [18–33]. Although higher protein expression seems to be associated with reduced time to recurrence [23, 34, 35] or progression [18, 19, 23, 26, 32, 34], but this association could not be demonstrated by several other authors (Table 1) [22, 24–26, 28, 30, 31]. Only Saint et al. (2004) [23] and Lee (1997) [35] found that p53 could be an independent prognostic factor, but with opposite results. TP53 gene mutation was also associated with higher recurrence rate [36]. It seems that p53 could not be a suitable predictive marker, since the majority of the studies could not corroborate these findings.

#### 3.1.2. Ki-67

Ki-67 is a nuclear protein for cellular proliferation, used as a marker of cell proliferation index. Higher ki-67 expression seems to be associated with recurrence after BCG [27, 31, 35, 37] and with lower time to recurrence [22, 34, 35]. Still, multivariate analysis failed to prove its value as an independent predictive marker [22, 34, 35]. Furthermore, Lopez-Beltran et al. [34] and Blanchet et al. [38] found that the Ki-67 expression could be associated with lower PFS in univariate analysis and multivariate analysis, respectively. At the moment, Ki-67 could not be used as predictive marker of BCG response, due to the fact that half of the studies regarding this marker did not find any association with BCG treatment response.

#### 3.1.3. (Retinoblastoma Protein) pRB 

Only three studies evaluated the tumor suppressor protein, pRB; namely, Cormio and colleagues in 2010 [39] assessed pRB-altered expression in only 27 patients treated with a full maintenance BCG treatment schedule (mBCG) and found it associated with RFS and PFS. Park et al. [26] and Esuvaranathan et al. [30] evaluate pRB in patients subjected only to induction schedule with BCG (iBCG) and did not find any relationship with protein-positive staining and recurrence, RFS or PFS. These findings suggest that this marker could be a possible indicator of BCG response in patients treated with mBCG although more studies need to be performed in order to clarify this association.

#### 3.1.4. CD68 (Marker of TAMs Presence)

Tumour-associated Macrophages (TAMs) may have a dual role in cancer. They could be involved in tumor-cell elimination or can stimulate tumor-cell proliferation, promote angiogenesis, and favour invasion and metastasis [40]. CD68 is a glycoprotein, and its expression allows identifying macrophages.In 2009 Ayari et al. [41] found that a higher TAM count in peritumoural region was associated with lower RFS and with a high risk of BCG treatment failure. The same was reported for CIS tumors treated with BCG by Takayama [42]. This marker could be a suitable biomarker for predicting BCG treatment response although more studies are necessary to confirm these findings and to prove TAMs influence in BCG immunotherapy response.

#### 3.1.5. Other Intracellular Markers

c-erB2 is a proto-oncogene, member of the epidermal growth factor receptor (EGFR/ErbB) family. Janane et al. (2011) [43] found that c-erB2 expression was associated with lower RFS after BCG treatment. Apoptosis regulator protein, bcl-2, was also studied, but doubts persist about its predictive value of BCG treatment outcome due to conflicting results found by Okamura et al. [20] and Lee et al. [35]. Some authors evaluated the role cyclin-dependent kinase inhibitors, p21 and 27, as predictors of BCG response. Zlotta et al. [22] found that higher p21 expression was associated with decreased RFS in univariate analysis, and Lopez-Beltran and colleagues [34] found that higher expression of p27 was associated with decreased RFS and PFS. These markers are regarded unsuitable candidates to predict BCG treatment response, due to the lack of consistency of the so far presented results (see Table 1). Proteins involved in cell cycle regulation, such as Cyclin D1 and D3, were found to be slightly associated to reduced RFS and PFS [34] although these results were limited to one study, thus needing further investigation. On the other hand, Cyclin D3 gene amplification was also associated with decreased RFS as shown by Lopez-Beltran et al. [44].

#### 3.1.6. Other Protein Markers

Other 30 different markers were also studied, as shown in Table 1. All of them were evaluated only in one single study. One of the most promising markers is ezrin, a cytoplasmic peripheral membrane protein involved in cell surface structure adhesion, migration, and organization. Palou et al. [31] never shown that this protein was associated to higher recurrence rate, reduced RFS and PFS. Other markers have shown some potential as predictive marker. Cox-2, which promotes the conversion of arachidonic acid to prostaglandins, could also help to predict early recurrence and progression [45]. Yutkin et al. [46] studied natural killer cells cytotoxic receptors and described that expression of Nkp family proteins, 30, 44, and 46, were associated with less recurrence after treatment. Heat shock protein 90 (HSP90) loss of expression was associated to higher recurrence and progression rates [47]. These may therefore be candidate markers to predict recurrence after BCG treatment.

 All of these markers, and others [41, 48] need further investigation once they were only evaluated in one study and with samples rounding 30 or 50 patients, almost only treated with iBCG schedule.

#### 3.1.7. Genetic Markers Evaluated on Tumour

**Gene Expression**

Other markers have been studied in tumour biopsies, such as genetic markers (not shown in Table 1). Gazzaniga et al. (2009) [49] evaluated *α*5*β*1 integrin gene expression (the integrin involved in BCG attachment and internalization into cells) in the tumours of 11 patients treated with BCG and found that lower *α*5*β*1 expression was associated with recurrence [49].

Videira et al. [50] evaluated the expression of 10 immunological genes involved in antigen presentation (CD1 and MHC-I) and chemokines (MIP-1, MCP-1/2, IP10 and MIG). This study showed higher mRNA levels of MHC-I for tumours that will not relapse after treatment and tumours that will recur have lower expression of CD1c, CD1e and MCP-1. They also found higher expression of CD1a, CD1b, CD1c, CD1e, MHC-I, MIG, and IP10 in biopsies after treatment in the group of patient without recurrence when compared with the recurrence group [50].

Kim and colleagues [51] performed a microarray analysis in tumours from 80 patients treated with BCG, and they could identify a subset of genes that individually are associated with reduced RFS and PFS. When evaluated together, the “poor predictive signature” presented a 3.38 higher risk of recurrence or 10,49 higher risk of progression after BCG treatment [51].

These findings demonstrate that evaluation of gene expression patterns in tumours prior to treatment has the potential to undisclose a new subset of biomarkers capable predicting BCG treatment response. More studies are needed to validate these markers and possible find new ones.

**Gene Methylation**

Alvarez-Múgica et al. [52] studied the methylation status of myopodin gene (involved in actin-bundling activity) and found that this event is associated with reduced RFS [52]. Recently, Agundez and colleagues [53] evaluated methylation status in 25 tumour suppressor genes. It was found that differential methylation for several genes had an impact BCG treatment outcome. Therefore, methylation of PAX6 gene is associated with lower RFS [53]. However, unmethylated MSH6, RB1, THBS1, PYCARD, TP73, ESR1, and GATA5 genes are associated with higher PFS [53]. This new approach could contribute to establish new candidate predictive biomarkers of BCG treatment response.

### 3.2. Urinary Markers

#### 3.2.1. IL-8 (Major Mediators of the Inflammatory Response)

Urinary levels of the chemokine IL-8, a potent chemoattractant of neutrophils and macrophages, could be a potential biomarker of BCG treatment response. Several authors found that higher IL-8 levels are significantly associated with a better treatment outcome [54–62]. Only Sagnak et al. (2009) [54] and Watanabe et al. (2003) [56] found that lower levels of IL-8 are a slightly associated with reduced RFS. These studies presented levels measured in different time points of BCG treatment and its predictive value was accessed with different cutoff values; therefore, it is imperative to evaluate the same cutoff values in larger sets of samples.

#### 3.2.2. Interleukin 2 (IL-2)

IL-2 is a Th1 subset cytokine, involved in cytotoxic T lymphocyte expansion (cytotoxic T lymphocytes and natural killer cells) and macrophage activation. IL-2 urinary levels were extensively studied [56, 58, 60, 63–66], and higher IL-2 urinary levels were appointed to be a good predictive marker of recurrence [56, 58, 60, 65, 66] and higher RFS [56, 63, 64]. Saint also found that lower or absent levels of IL-2 were associated with shorter PFS in mBCG-treated patients but not in iBCG [63, 64]. IL-2 urinary levels are the most promising predictive biomarker of BCG treatment response; however, it could only be measured during treatment and could not be used in treatment definition. These results highlight the key role of IL-2 in BCG treatment response; therefore, it is important to evaluate why nonresponders have lower IL-2 levels, in order to establish IL-2-related biomarkers that could predict BCG response prior to treatment.

#### 3.2.3. Other Urinary Cytokines

Other urinary cytokines have demonstrated to have potential as predictive biomarkers, yet some need further investigation. Tumour necrosis factor *α* (TNF-*α*), whose primary role is the regulation of immune cells, and its urinary levels have been evaluated during the course of BCG treatment in several studies. It was found that higher TNF-*α* levels are associated with a higher response rate [55, 56, 58, 60, 66]. Watanabe et al. (2003) [56], also demonstrated that higher levels of this molecule are associated with better RFS.

IL-6 is an interleukin that acts as both a proinflammatory and anti-inflammatory cytokine. It is secreted by T cells and macrophages to stimulate immune response. Higher IL-6 urinary levels during BCG treatment were associated with lower recurrence rates and higher RFS [56, 58, 60, 66].

IL-18 is a proinflammatory cytokine, produced by macrophages, and induces cell-mediated immunity. Lower urinary levels of this protein have been found within the first 12 h after BCG in nonresponders to BCG treatment [59]. Although this cytokine was only evaluated in 17 patients, others authors suggest that IL-18 has a key role in the mechanism of intravesical immunotherapy with BCG [67].

IFN-*γ* is involved in macrophage activation and Th1 differentiation, and higher urinary levels were associated with a good treatment response in a first course of iBCG [65], yet other authors could not confirm this association [55, 56, 60, 64].

Granulocyte-macrophage colony-stimulating factor (GM-CSF) is a cytokine that functions as a white blood cell growth factor. GM-CSF stimulates stem cells to produce granulocytes (neutrophils, eosinophils, and basophils) and monocytes. GM-CSF levels were evaluated in 2 papers [55, 60]; only Jackson et al. (1998) [60] found that higher levels of these molecule were associated with reduced recurrence rate.

Somehow, all of these cytokine are associated with treatment response; however, their predictive value fails to be consistent among the studies. Once more, important molecules involved in BCG mechanism of action have been highlighted; hence, it is essential to explore other biomarkers related to these cytokine urinary levels variability.

#### 3.2.4. Other Markers

Other 7 markers were evaluated in 4 papers, only regarding recurrence rate [60, 68, 69]. Higher levels of survivin (member of the inhibitor of apoptosis family) and soluble CD14 (acts as a coreceptor in recognize pathogen-associated molecular patterns) were present in the recurrence group [60, 68]. The soluble intercellular adhesion molecule 1 (ICAM-1), which facilitates transmigration of leukocytes across vascular endothelia in processes such as extravasation and the inflammatory response, was associated with recurrence in multivariate analysis [60]. The biomarker value of these molecules warrants further studies in order to evaluate its role in BCG immunotherapy response.

Efforts were made in order to find serological predictive markers of BCG treatment outcome. Molecules such as purified protein derivative (PPD), HSP65/70, major secreted antigen complex (Ag85), immunogenic, and skin-reactive protein, p64, have been explored [70–72]. Still, the serological levels of these proteins were not able to predict BCG treatment failure [70–72]. Also, several immunological mediators were evaluated in blood of BCG-treated patients, but none was associated with recurrence after BCG treatment with the exception that lower levels of IL-2 appear to be associated with recurrence [70, 71]. Therefore, with the exception of IL-2, molecules found in the peripheral circulation may not be a suitable approach to find predictive biomarkers of BCG response.

### 3.3. Genetic Polymorphisms

#### 3.3.1. *NRAMP1*(SLC11A1) Gene

*Natural resistance-associated macrophage protein 1 (NRAMP1)* gene regulates intracellular pathogen proliferation and macrophage inflammatory responses. *NRAMP1* is one of the most studied genes, with 5 polymorphisms analyzed in 2 papers [73, 74]. Chiong et al.(2010) [73] found that (GT)n repeat and D543N GA genotype were associated with reduced RFS, this author also studied *hGPX1* gene, and an association was found [73]. On the other hand, Decobert in 2006 [74] found that D543N GG genotype is also associated with reduced time to recurrence.

#### 3.3.2. DNA Repair Genes

Gu et al. (2005) [75] analyzed several polymorphisms in *XPA, XPC, XPD, XPG, ERCC1,* and *ERCC6* genes and found that *XPA* 5′UTR AA was correlated with higher RFS when compared with AG and GG genotypes, and *ERCC6* Met1097Val GG genotype was associated with reduced RFS after BCG treatment [75]. However, Gangwar and colleagues (2010) [76] have also studied *XPC* gene polymorphisms and found that patients carrying AC or CC genotypes of *XPC* Lys939Gln have reduced RFS [76]. The same author published other paper in 2010 regarding polymorphisms in *APEX1* and *ERCC2* genes and found that *ERCC2* Asp312Asn AA was also associated with reduced RFS [77]. Polymorphisms in *XRCC*1/3/4 genes were also studied, only *XRCC1* codon11 AA genotype was associated with reduced RFS after BCG treatment [78, 79].

#### 3.3.3. Inflammation-associated Genes

Rama Mittal group has published several studies [80–83] regarding several polymorphisms in inflammatory genes such as *IFNG, TNFA, TGFB1, COX2, PPARG, IL1B, IL1RN, IL4, IL6,* and *IL8* [80, 81, 83–85]. They found that *IL8-251* AA, *TNFA-1031* CC, *IL6-174* CC, and *TGFB1*+28 TT genotypes were associated with higher RFS after BCG treatment [80, 81, 84, 85]. On the other hand, they found that patients carrying *COX2-765* CC genotype or NFKB ATTG Del/Del genotypes or *IFNA* LOH or *IFNG*+874 A allele have a decreased RFS after treatment. Considering the *IL6-174* G/C, Leibovici et al.(2005) [86] found conflicting results in which CC genotype was associated with a reduced RFS after BCG. Other paper (not shown in Table 3) evaluated the influence 22 polymorphisms in 13 inflammatory genes on recurrence after BCG treatment [87]; patients carrying the *TGFB* codon 10 T allele, *TGFB* codon 25 G allele, *IL4-1098* GG genotype, and *IL10-1082* GG genotype are at higher risk of recurrence after BCG treatment [87].

#### 3.3.4. Cell Cycle and Apoptosis Genes

The role of genetic polymorphisms on genes such as *MMP1/2/3/7/8/9, FAS, CASP8/9, MDM2,* and *CCDN1* on BCG treatment outcome was addressed by some authors [88–92]. It was found that patients carrying *MMP2-1306* T allele or *MMP3-1171* 5A/6A have a reduced RFS after treatment [91, 92] and patients carrying *MMP1-1607* 1G/2G or *CASP9-1263* GG or *MDM2*+309GG genotypes have an increased RFS [88–90].

#### 3.3.5. Sonic Hedgehog Pathway Genes

 A recent paper evaluated 177 polymorphisms (haplotype tag SNPs) in 11 genes on Sonic Hedgehog Pathway (Shh) [93]. The main result regarding BCG-treated patients shows that 2 polymorphisms in *GLI3* gene (rs6463089 and rs3801192) were associated with worse treatment outcome [93]. Patients carrying at least on variant allele of these SNPs have a decreased RFS when compared with wild-type carriers [93].

## 4. Discussion

 Several studies were conducted to personalize and improve the NMIBC treatment with BCG. A plethora of exciting data has emerged recently, which represents a potential tool to define differences in BCG treatment response.

Among the proteins associated with bladder cancer progression, p53 and ki67 are the most well studied. Still, the evaluation of these markers in the context of BCG treatment did not offer strong evidences regarding their role as predictive biomarkers.

 Conversely, CD68 has shown a huge potential as a predictive biomarker. Indeed, tumour-associated macrophages (TAMs), when detected at tumour core and surrounding tissue, strongly correlated with tumour treatment response [41, 42]. It has been suggested that a higher number of TAMs can promote a more efficient phagocytosis and elimination of BCG, preventing BCG from inducing a long-term local inflammation [41]. Although the results regarding this marker are consistent, complementary information are still necessary to confirm the predictive value of these marker and the influence of TAMs presence in the treatment outcome. Namely, it will be important to verify the phenotypic nature of this TAMS, as only the M2 macrophages are known to produce protumor factors such as inflammatory cytokines that could inhibit BCG treatment response [40].

Tumour markers like ezrin, HSP90, CD83, and others also reveal a potential as biomarkers of BCG treatment response. However, only one paper addresses these biomarkers in the context of BCG treatment outcome. In this sense, more studies are needed to validate if these markers are suitable candidates to predict BCG treatment outcome.

Urinary markers are widely studied worldwide, and several molecules, such as IL-8, IL-2, and, in a lesser extent, TNF-*α* and IL-18, are currently believed to play a role on BCG immunotherapy mechanism of action. More importantly, their levels have appeared to be associated with treatment failure. However, as state by Zuiverloon et al. [94], these markers are “during BCG markers,” only present in urine during the course of treatment, thus failing to provide insight on the outcome prior to that. pm Nonetheless, the role of urine in noninvasive approaches to monitor response has been demonstrated.

Pharmacogenomic investigation has also demonstrated to be a powerful tool in the identification of predictive biomarkers. Regarding BCG immunotherapy, several polymorphisms in a large set of genes have demonstrated the potential to predict treatment outcome. Polymorphism in inflammatory genes such as *IL8*, *TNFA*, *IL6*, *TGFB1, COX2,* and *IFNG* are examples of putative predictive markers [80, 81, 83–85].

However most of them were studied in the same Indian population which was small in number (80 patients). Moreover, the majority of these patients have been subjected only to a induction schedule with BCG (iBCG) [76–81, 83–85, 88–92]. In order to be used as a predictive markers,it is still necessary to evaluate these polymorphism in larger sets of patients, with a representative number of patients treated with a full maintenance BCG treatment schedule (mBCG) and from other ethnicities. Furthermore, there are several other molecules involved in BCG immunotherapy mechanism of action and potentially involved in the treatment response that may be subjected to polymorphism analysis. In a recent review, Alexandroff and colleagues suggest that molecules such as IL-2, IL-17, IL-23, soluble CD40L, and TRAIL may be important key targets and may serve as putative markers [16]. A careful evaluation of such candidates should be undertaken in order to access their biomarker value.

Recently, the studies by Kim and colleagues [51] using a microarray analysis allowed to identify a “poor predictive signature” of BCG treatment response. This work is suggesting that a combinatory analysis involving all predictive markers may permit to create a useful score or a predictive profile. The combination of several markers will allow explaining and consequently predicting all recurrences after BCG treatment. Other current approaches, such as microRNAs profiling and Genome wide association studies (GWAS) can be important features in the context of BCG immunotherapy research and treatment response prediction.

## 5. Conclusion

Regarding the tumour molecular characteristics studied, three major conclusions can be drawn, p53 and ki-67 are not suitable predictive biomarkers, markers such as TAMs and other molecules (ezrin, HSP90, CD83, and Cox2) require validation, and different approaches such as gene expression and epigenetic alterations of the tumour prior to treatment may bring new insights in the search for predictive biomarkers of BCG immunotherapy.

Concerning urinary markers, the monitoring of IL-2 levels during treatment seems a consistent noninvasive approach to determine treatment response; hence, other cytokines could have the same predictive power. The only drawback is the fact that these markers are unable to predictive treatment response prior to therapy.

In relation to genetic polymorphisms, those in the genes *IL8*, *TNFA*, *IL6*, *TGFB1, COX2,* and *IFNG* were found to be among the most informative. Nevertheless, it is important to validate the findings in larger samples from different ethnicities and evaluate other genetic polymorphism in molecules that have shown to have a important role in BCG immunotherapy mechanism of action (e.g., IL-2, TRAIL, and Th17 cytokines).

It is our belief that only the introduction of an array of biomarkers can improve the accuracy of current status on the prediction of BCG treatment outcome and thus improve the management of high-risk NMIBC. Future studies combining the most promising putative biomarkers are warranted if not mandatory.

## Figures and Tables

**Table 1:** Tumour-associated markers predicting BCG treatment outcome. The markers are ordered from the most studied to the less, and, within each marker, the studies are ordered by quality score.

Marker	Author	Quality	*n*	Treatment scheme	Impact	Outcome			
						Rec(*P*)	RFS (*P*/HR (95% CI))	Prog(*P*)	PFS (*P*/HR (95% CI))
p53									
	Lopez-Beltran et al., [34]	8/8	51	iBCG	−	X	0.0332/NS^∗^	X	0.0041/1.003 (1.002–1.074)
	Park et al., [26]	7/8	61	iBCG	−	NS	NS	X	0.0495
	Zlotta et al., [22]	7/8	47	iBCG	None	NS	NS/NS^∗^	NS	NS/NS^∗^
	Lee et al., [35]	7/8	32	iBCG	−	X	0.0027/3.8 (1.3–11.4)^∗^	X	X
	Lacombe et al., [18]	7/8	98	iBCG	−	NS	X	X	0.0001/2.5 (1.1–5.5)^∗^
	Palou et al., [31]	6/8	92	iBCG	<PFS-M	NS	NS	X	NS/0.018^∗^
	Esuvaranathan et al., [30]	6/8	80	iBCG	None	NS	NS	X	NS
	Kyroudi-Voulgari et al., [27]	6/8	66	iBCG	None	NS	X	X	X
	Cormio et al., [32]	5/8	27	mBCG	−	NS	NS	NS	0.06
	Saint et al., [23]	5/8	102	iBCG/mBCG	−	NS	0.03/0.15 (0.06–0.42)^∗^	0.001	<0.0001
	Peyromaure et al., [24]	5/8	29	iBCG	None	NS	NS	NS	NS
	Caliskan and Türkeri [19]	5/8	30	iBCG	>Prog	NS/NS^a^	X	NS/NS^a^	0.04
	Pages et al., [25]	4/8	43	iBCG	None	NS	NS	X	X
	Okamura et al., [20]	4/8	38	mBCG	None	NS	X	X	X
	Moyano Calvo et al., [29]	3/8	51	iBCG	None	NS	X	X	X
	Moyano Calvoet al., [33]	3/8	71	iBCG	None	NS	X	NS	X
	Lebret et al., [21]	3/8	35	iBCG	None	NS	X	X	X
	Serdar et al., [28]	1/8	24	iBCG	None	NS	NS	X	X

Ki-67									
	Lopez-Beltran et al., [34]	8/8	51	iBCG	−	X	0.0034/NS^∗^	X	0.0163/NS^∗^
	Park et al., [26]	7/8	61	iBCG	−	NS	NS	X	NS
>25%	Zlotta et al., [22]	7/8	47	iBCG	−	NS	0.02/NS^∗^	NS	NS/NS^∗^
	Lee et al., [35]	7/8	32	iBCG	−	0.0413	0.0164/NS^∗^	X	X
	Palou et al., [31]	6/8	92	iBCG	>Rec	0.015	NS	X	NS/NS^∗^
	Kyroudi-Voulgari et al., [27]	6/8	66	iBCG	−	<0.05	X	X	X
	Blanchet et al., [38]	5/8	57	iBCG	−	X	NS/NS^∗^	X	0.0001/4.61 (*P* < 0.04)
>20%	Lebret et al., [37]	5/8	25	iBCG	−	0.03	X	X	X
	Moyano Calvo et al., [33]	3/8	71	iBCG	None	NS	X	NS	X
	Moyano Calvo et al., [29]	3/8	51	iBCG	None	NS	X	X	X
pRB									
	Park et al., [26]	7/8	61	iBCG	−	NS	NS	X	NS
+	Esuvaranathan et al., [30]	6/8	80	iBCG	None	NS	NS	X	NS
Altered exp	Cormio et al., [39]	5/8	27	mBCG	−	X	0.037	X	0.018

CD68									
High TAM	Ayari et al., [41]	6/8	46	iBCG/mBCG	−	X	0.093/3.81 (1.32–11)^b^	X	X
High TAM	Takayama et al., [42]	6/8	41^1^	iBCG	−	0.0023	0.0002/1.7 (1.48–5.03)^c^	X	X
	Kitamura et al., [48]	4/8	30	iBCG	None	X	NS/NS^∗^	X	X

c-erbB2	Lee et al., [35]	7/8	32	iBCG	None	X	NS/NS^∗^	X	X
	Janane et al., [43]	5/8	84	iBCG	−	X	<0.01	X	X
	Morgan et al., [95]	5/8	82	iBCG	None	NS	X	X	X

E-Cadherin	Moyano Calvo et al., [29]	3/8	51	iBCG	None	NS	X	X	X
	Serdar et al., [28]	1/8	24	iBCG	None	NS	NS	X	X

bcl-2	Lee et al., [35]	7/8	32	iBCG	−	X	0.0112/NS^∗^	X	X
	Okamura et al., [20]	4/8	38	mBCG	+	0.044	X	X	X

p21	Lopez-Beltran et al., [34]	8/8	51	iBCG	None	X	NS/NS^∗^	X	NS/NS^∗^
>10%	Zlotta et al., [22]	7/8	47	iBCG	−	NS	0.02/NS^∗^	NS	NS/NS^∗^

p27	Lopez-Beltran et al., [34]	8/8	51	iBCG	+	X	0.0005/0.997 (0.995–0.999)^∗^	X	0.0161/NS^∗^
	Park et al., [26]	7/8	61	iBCG	−	NS	NS	X	NS

Cyclin D1	Lopez-Beltran et al., [34]	8/8	51	iBCG	−	X	0.0103/NS^∗^	X	<0.0001/1.009 (1.002–1.074)^∗^

Cyclin D3	Lopez-Beltran et al., [34]	8/8	51	iBCG	−	X	0.0332/NS	X	0.0041/1.003 (1.002–1.074)^∗^

PTEN	Park et al., [26]	7/8	61	iBCG	−	NS	NS	X	NS

FGFR3	Park et al., [26]	7/8	61	iBCG	−	NS	NS	X	NS

CD9	Park et al., [26]	7/8	61	iBCG	−	NS	NS	X	NS

hTERT									
Pre-treat >75%	Zachos, [96]	7/8	30	iBCG	−	X	0.05/NS^c^	NS/NS^c^	X

c-myc	Lee et al., [35]	7/8	32	iBCG	None	X	NS/NS^∗^	X	X

Cathepsin D	Lee et al., [35]	7/8	32	iBCG	−	X	0.0235/NS^∗^	X	X

CD83									
High CD83+	Ayari et al., [41]	6/8	53	mBCG	−	X	0.0001/9.81 (1.12–85.7)^d^	X	X

Ezrin	Palou et al., [31]	6/8	92	iBCG	−	0.041	0.06	X	0.009/0.031^∗^

NKp30	Yutkin, [46]	6/8	17	iBCG	<Rec	0.0026	X	X	X

NKp44	Yutkin, [46]	6/8	17	iBCG	<Rec	0.027	X	X	X

NKp46	Yutkin, [46]	6/8	17	iBCG	<Rec	0.044	X	X	X
PD-L1	Inman et al., [97]	5/8	44	iBCG/mBCG	None	NS	X	X	X

CD25	Honda et al., [98]	5/8	16	iBCG	None	NS	X	X	X

Cox-2	Kim et al., [45]	5/8	37	iBCG	−	X	0.0493	X	0.0272

VEGF	Morgan et al., [95]	5/8	82	iBCG	None	NS	X	X	X

TCR *γ*/*δ*	Honda et al., [98]	5/8	16	iBCG	None	NS	X	X	X

HSP60	Lebret et al., [47]	4/8	33	iBCG	None	NS	X	NS	X

HSP90									
Loss exp	Lebret et al., [47]	4/8	33	iBCG	−	0.0001	X	0.0001	X

CD4	Kitamura et al., [48]	4/8	30	iBCG	None	X	NS/NS^∗^	X	X

CD8	Kitamura et al., [48]	4/8	30	iBCG	None	X	NS/NS^∗^	X	X

HLA class I	Kitamura et al., [48]	4/8	30	iBCG	+	X	0.0394/0.06 (0.01–0.4)	X	X

CD20	Kitamura et al., [48]	4/8	30	iBCG	None	X	NS/NS^∗^	X	X

TIA-1	Kitamura et al., [48]	4/8	30	iBCG	None	X	0.0393/NS^∗^	X	X

S-100	Kitamura et al., [48]	4/8	30	iBCG	None	X	NS/NS^∗^	X	X

FOXP3	Kitamura et al., [48]	4/8	30	iBCG	None	X	NS/NS^∗^	X	X

PCNA	Okamura et al., [20]	4/8	38	mBCG	None	NS	X	X	X

HSP65	Ardelt et al., [99]	3/8	16	mBCG	None	NS	X	X	X

B-Catenin	Moyano Calvo et al., [29]	3/8	51	iBCG	+	<0.05	X	X	X

−: negative impact, marker associated with a poor BCG response.

+: positive impact, marker associated to a better BCG response.

Rec: recurrence; *P* value for recurrence.

RFS: recurrence-free survival; *P* value for log-rank test/HR: hazard ratio from Cox regression; (95% Cl): 95% confidence interval.

Prog: progression; *P* value for progression.

PFS: progression-free survival; *P* value for log-rank test/HR: hazard ratio from Cox regression; (95% Cl): 95% confidence interval.

iBCG: induction BCG scheme only.

mBCG: maintenance BCG scheme.

NS: no statistical significance.

X: not evaluated.

^∗^all analysed variables (independent prognostic factor).

^a^adjusted for grade and stage.

^b^adjusted for age, gender, T stage and number of mBCG instillations.

^c^adjusted for age and gender.

^d^adjusted for age, gender, T stage.

^1^only CIS patients.

**Table 2:** Urinary markers predicting BCG treatment outcome. The markers are ordered from the most studied to the less, and, within each marker, the studies are ordered by quality score.

Marker	Author	Quality	*n *	Treatment scheme	Impact	Outcome			
						Rec(*P*)	RFS (*P*/HR (95% CI))	Prog(*P*)	PFS (*P*/HR (95% CI))
IL-8	Sagnak et al., [54]	6/8	41	iBCG	−	X	0.006/2.98 (1.02–8.72)^a^	X	X
	Kumar et al., [57]	5/8	26	iBCG	+	0.001	X	X	X
	Sanchez-Carbayo et al., [58]	5/8	15	iBCG	None	NS	X	X	X
	Jackson et al., [60]	5/8	34	iBCG	None	NS/NS^∗^	X	X	X
	Rabinowitzir et al., [62]	5/8	46	iBCG	None	NS	X	X	X
	Shintani et al., [55]	4/8	20	iBCG	None	NS	X	X	X
	Watanabe et al., [56]	4/8	20	iBCG	+	<0.05	0.013/NS^∗^	X	X
	Thalmann et al., [59]	4/8	17	iBCG	+	0.0209	X	X	X
	Thalmann et al., [61]	4/8	20	iBCG	+	0.0002	X	X	X

IL2	Saint et al., [64]	5/8	37	iBCG	+	X	0.0009	X	NS
	Sanchez-Carbayo et al., [58]	5/8	15	iBCG	+	0.041	X	X	X
	Jackson, [60]	5/8	34	iBCG	None	NS/NS^∗^	X	X	X
	De Reijke et al., [66]	5/8	23	iBCG	+	0.003	X	X	X
	Saint et al., [63]	4/8	39	mBCG	+	X	0.01	X	0.01
	Watanabe et al., [56]	4/8	20	iBCG	+	<0.01	0.0003/0.37 (0.03–0.895)^∗^	X	X
	Saint et al., [65]	4/8	19	iBCG + iBCG	None/+	NS/ <0.05	X	X	X

IFN-*γ*	Saint et al., [64]	5/8	37	iBCG	None	X	NS	X	NS
	Jackson, [60]	5/8	34	iBCG	None	NS/NS^∗^	X	X	X
	Shintani et al., [55]	4/8	20	iBCG	None	NS	X	X	X
	Watanabe et al., [56]	4/8	20	iBCG	None	NS	NS/NS^∗^	X	X
	Saint et al., [65]	4/8	19	iBCG + iBCG	+/None	<0.05/ NS	X	X	X

TNF-*α*	Sanchez-Carbayo et al., [58]	5/8	15	iBCG	None	NS	X	X	X
	Jackson, [60]	5/8	34	iBCG	+	NS/ <0.05^∗^	X	X	X
	De Reijke et al., [66]	5/8	23	iBCG	+	0.025	X	X	X
	Shintani et al., [55]	4/8	20	iBCG	None	NS	X	X	X
	Watanabe et al., [56]	4/8	20	iBCG	+	<0.05	0.012/NS^∗^	X	X
IL-10	Saint et al., [64]	5/8	37	iBCG	None	X	NS	X	NS
	Jackson, [60]	5/8	34	iBCG	None	NS/NS^∗^	X	X	X
	Saint et al., [63]	4/8	39	mBCG	None	X	NS	X	NS
	Watanabe et al., [56]	4/8	20	iBCG	+	<0.01	0.009/NS^∗^	X	X
	Saint et al., [65]	4/8	19	iBCG + iBCG	None	NS	X	X	X

IL-6	Sanchez-Carbayo et al., [58]	5/8	15	iBCG	None	NS	X	X	X
	Jackson, [60]	5/8	34	iBCG	None	NS/NS^∗^	X	X	X
	De Reijke et al., [66]	5/8	23	iBCG	+	0.04	X	X	X
	Watanabe et al., [56]	4/8	20	iBCG	+	<0.05	0.023/NS^∗^	X	X

Urovysion (FISH)	Whitson et al., [100]	6/8	48	mBCG	−	X	<0.01/6.7 (2.1–22.1)^∗^	X	X
+posttreat	Savic et al., [101]	5/8	68	iBCG	−	X	<0.001/5.6 (2.5–12.2)^∗^	X	X
	Mengual, [102]	5/8	65	iBCG	−	X	0.015/2.7(1.18–6.15)^∗^	X	X
+pretreat	Kipp et al., [103]	5/8	37	iBCG	−	X	NS/3.3(1.3–8.5)^∗^	X	NS/NS^∗^
+posttreat					−	X	<0.001/4.6 (1.9–11.1)	X	0.001/9.4 (1.9–45.3)

IL-12	Jackson, [60]	5/8	34	iBCG	None	NS/NS^∗^	X	X	X
	Shintani et al., [55]	4/8	20	iBCG	None	NS	X	X	X
	Watanabe et al., [56]	4/8	20	iBCG	None	NS	NS/NS^∗^	X	X

IL-1*β*	De Reijke et al., [66]	5/8	23	iBCG	None	NS	X	X	X
	Shintani et al., [55]	4/8	20	iBCG	None	NS	X	X	X
	Watanabe et al., [56]	4/8	20	iBCG	None	NS	NS/NS^∗^	X	X

GM-CSF	Jackson, [60]	5/8	34	iBCG	−	<0.05/ <0.05^∗^	X	X	X
	Shintani et al., [55]	4/8	20	iBCG	None	NS	X	X	X

WBC	Saint et al., [104]	5/8	72	mBCG	+	X	0.009	X	X
	Shintani et al., [55]	4/8	20	iBCG	None	NS	X	X	X

G-CSF	Shintani et al., [55]	4/8	20	iBCG	None	NS	X	X	X

FN	Danişman et al., [69]	5/8	38	iBCG	None	NS	X	X	X

IL-4	Jackson, [60]	5/8	34	iBCG	None	NS/NS^∗^	X	X	X

sICAM-1	Jackson, [60]	5/8	34	iBCG	+	NS/ <0.05^∗^	X	X	X
sCD14	Jackson, [60]	5/8	34	iBCG	−	NS/ <0.05^∗^	X	X	X

Survivin	Hausladen, [68]	4/8	23	iBCG	−	<0.05	X	X	X

IL-18	Thalmann et al., [59]	4/8	17	iBCG	+	0.0464	X	X	X

iBCG: induction BCG scheme only.

mBCG: maintenance BCG scheme.

−: negative impact, marker associated with a poor BCG response.

+: positive impact, marker associated to a better BCG response.

Rec: recurrence; *P* value for recurrence.

RFS: recurrence-free survival; *P* value for log-rank test/HR: hazard ratio from Cox regression; (95% Cl): 95% confidence interval.

Prog: progression; *P* value for progression.

PFS: progression-free survival; *P* value for log-rank test/HR: hazard ratio from Cox regression; (95% Cl): 95% confidence interval.

NS: no statistical significance.

X: not evaluated.

^∗^all analysed variables (indepent prognostic factor).

^a^adjusted for BCG-related complications, tumour stage, and grade.

**Table 3:** Genetic polymorphisms associated to BCG outcome. The markers are ordered from the most studied to the less, and, within each marker, the studies are ordered by quality score.

Marker	Author	Quality	*n*	Treatment scheme	Impact	RFS (*P*/HR (95% CI))
*NRAMP*						
D543N GG	Chiong et al., [73]	6/8	99	mBCG	−	0.033/4.6 (1.4–15.2)^a^
D543N GA	Decobert et al., [74]	6/8	67	iBCG + mBCG	−	0.0271/5.74 (2.4–13.8)^b^
(GT)*n* allele 3	Chiong et al., [73]	6/8	99	mBCG	−	NS/24.8 (3.08–199.9)^a^
	Decobert et al., [74]	6/8	67	iBCG + mBCG	None	NS/NS^b^
469 + 14 G/C	Decobert et al., [74]	6/8	67	iBCG + mBCG	None	NS/NS^b^
274 C/T	Decobert et al., [74]	6/8	67	iBCG + mBCG	None	NS/NS^b^
1465 − 85 G/A	Decobert et al., [74]	6/8	67	iBCG + mBCG	None	NS/NS^b^

*XPA* 5^′^UTR A/G	Gu et al., [75]	6/8	112	iBCG + mBCG	−	0.078

*XPC*						
Lys 939 Gln	Gangwar et al., [76]	7/8	77	iBCG	−	0.044/3.98 (1.02–10.7)^∗^
	Gu et al., [75]	6/8	112	iBCG + mBCG	None	NS
PAT ins/del	Gangwar et al., [76]	7/8	77	iBCG	None	NS^∗^
	Gu et al., [75]	6/8	112	iBCG + mBCG	None	NS
Ala 499 Val	Gu et al., [75]	6/8	112	iBCG + mBCG	None	NS

*XPD*						
Asp312Asn	Gu et al., [75]	6/8	112	iBCG + mBCG	None	NS
Lys751Gln	Gu et al., [75]	6/8	112	iBCG + mBCG	None	NS

*XPG* Asp1104His	Gu et al., [75]	6/8	112	iBCG + mBCG	None	NS

*IL8*						
−251 T/A	Ahirwar et al., [81]	7/8	71	iBCG	+	<0.001/0.12 (0.04–0.38)^c^
	Leibovici, [86]	6/8	123	iBCG/mBCG	None	NS^d^/NS^d^
+678 C/T	Ahirwar et al., [81]	7/8	71	iBCG	None	NS^c^

*TNFA*						
−1031 T/C	Ahirwar et al., [85]	7/8	73	iBCG	+	0.024/0.38 (0.14–0.98)^c^
−857 C/T	Ahirwar et al., [85]	7/8	73	iBCG	None	NS^c^
−863 C/A	Ahirwar et al., [85]	7/8	73	iBCG	None	NS^c^
−308 G/A	Ahirwar et al., [84]	6/8	69	iBCG	None	NS^e^
	Leibovici, [86]	6/8	123	iBCG/mBCG	None	NS^d^/NS^d^

*IL6* −174 G/C	Ahirwar et al., [84]	6/8	69	iBCG	+	0.021/0.298 (0.09–091)^e^
	Leibovici, [86]	6/8	123	iBCG/mBCG	−	NS^d^/4.6 (1.24–17)^d^

*hGPX* Pro198Leu C/T	Chiong et al., 2010 [73]	6/8	99	mBCG	None	NS^a^

*MMP1*						
−519 A/G	Srivastava, [90]	6/8		iBCG	None	NS
−1607 1G/2G	Srivastava, [90]	6/8		iBCG	+	0,030

*MMP2*						
−735 C/T	Srivastava, [91]	7/8	78	iBCG	None	NS^c^
−1306 C/T	Srivastava, [91]	7/8	78	iBCG	−	0.039/2.06 (1.01–4.18)^c^

*MMP3*						
−1171 5A/6A	Srivastava, [92]	6/8	78	iBCG	−	0.025/2.01 (0.98–4.12)^c^
Rs6796720 G/A	Srivastava, [92]	6/8	78	iBCG	None	NS^c^
Rs 520540 A/G	Srivastava, [92]	6/8	78	iBCG	None	NS^c^

*MMP7* −181 A/G	Srivastava, [90]	6/8		iBCG	None	NS

*MMP8* +799 C/T	Srivastava, [91]	7/8	78	iBCG	None	NS^c^

*MMP9*						
Q279R A/G	Srivastava, [92]	6/8	78	iBCG	None	NS^c^
P574R G/C	Srivastava, [92]	6/8	78	iBCG	None	NS^c^
R668Q G/A	Srivastava, [92]	6/8	78	iBCG	None	NS^c^
*ERCC1* 3^′^UTR G/T	Gu et al., [75]	6/8	112	iBCG/mBCG	None	NS

*ERCC2*						
Asp312Asn G/A	Gangwar et al., [77]	6/8	74	iBCG	−	0.005/3.07 (1.22–7.68)^c^
Lys751Gln A/C	Gangwar et al., [77]	6/8	74	iBCG	None	NS^c^

*ERCC6*						
Met1097Val A/G	Gu et al., [75]	6/8	112	iBCG/mBCG	−	0.022
Arg1230Pro G/C	Gu et al., [75]	6/8	112	iBCG/mBCG	None	NS

*APEX1* Asp148Glu T/G	Gangwar et al., [77]	6/8	74	iBCG	None	NS^c^

*COX2*						
−1290 A/G	Gangwar et al., [83]	6/8	79	iBCG	None	NS^e^
−1195 G/A	Gangwar et al., [83]	6/8	79	iBCG	None	NS^e^
−765 G/C	Gangwar et al., [83]	6/8	79	iBCG	−	2.43 (0.34–1.85)^e^
+8473 T/C	Gangwar et al., [83]	6/8	79	iBCG	None	NS^e^

*IFNA* LOH	Cai, [82]	7/8	77	mBCG	−	<0.0001/4.09 (2.59–6.28)^∗^

*IFNG* +874 T/A	Ahirwar et al., [80]	7/8	73	iBCG	−	2.24 (1.06–5.80)^c^

*NFkB* ATTG Ins/Del	Ahirwar et al., [81]	7/8	71	iBCG	−	0.031/2.53 (1.00–6.36)^c^

*CASP9*						
−1263 A/G	Gangwar et al., [88]	7/8	79	iBCG	+	0.024/0.27 (0.15–0.62)^c^
−293 Ins/Del	Gangwar et al., [88]	7/8	79	iBCG	None	NS^c^

*CASP8* −6N Ins/Del	Gangwar et al., [88]	7/8	79	iBCG	None	NS^c^

*IL4* VNTR	Ahirwar et al., [84]	6/8	69	iBCG	None	NS^e^

*IL1B* −511 C/T	Ahirwar et al., [80]	7/8	73	iBCG	None	NS^c^

*IL1RN* VNTR	Ahirwar et al., [80]	7/8	73	iBCG	None	NS^c^

*TGFB1* +28 C/T	Ahirwar et al., [80]	7/8	73	iBCG	+	0.37 (0.14–0.98)^c^

*MDM2* +309 G/T	Gangwar et al., [89]	6/8	79	iBCG	+	0.25 (0.08–0.80)^c^

*CCDN1* +870G/A	Gangwar et al., [89]	6/8	79	iBCG	None	NS^c^

*FAS* −670A/G	Gangwar et al., [89]	6/8	79	iBCG	None	NS^c^

*XRCC1*						
Arg194Trp C/T	Mittal et al., [78]	5/8	61	iBCG	None	NS^∗^
Arg280His G/A	Mittal et al., [78]	5/8	61	iBCG	None	NS^∗^
Arg399Gln G/A	Mittal et al., [78]	5/8	61	iBCG	−	0.004/5.05 (1.34–19.01)^∗^

*XRCC3*						
+18067 C/T	Mittal et al., [79]	7/8	73	iBCG	None	NS^c^
+17893 A/G	Mittal et al., [79]	7/8	73	iBCG	None	NS^c^

*XRCC4*						
+1394 G/T	Mittal et al., [79]	7/8	73	iBCG	None	NS^c^
Intron 3 (rs2836007)	Mittal et al., [79]	7/8	73	iBCG	None	NS^c^
Intron 7 (rs2836317)	Mittal et al., [79]	7/8	73	iBCG	None	NS^c^
Intron 7 (rs1805377)	Mittal et al., [79]	7/8	73	iBCG	None	NS^c^

*PPARG* Pro12Ala	Leibovici, [86]	6/8	123	iBCG/mBCG	None	NS^d^/NS^d^

*GLI3*						
rs6463089 G/A	Chen et al., [93]	7/8	204	iBCG + mBCG	−	2.40 (1.50–3.84)^∗^
rs3801192 G/A	Chen et al., [93]	7/8	204	iBCG + mBCG	−	2.54 (1.47–4.39)^∗^

iBCG: induction BCG scheme only.

mBCG: maintenance BCG scheme.

−: negative impact, marker associated with a poor BCG response.

+: positive impact, marker associated to a better BCG response.

RFS: recurrence-free survival; *P* value for log-rank test/HR: hazard ratio from Cox regression; (95% Cl): 95% confidence interval.

NS: no statistical significance.

^∗^all analysed variables (indepent prognostic factor).

^a^adjusted for age, gender, ethnicity, tumour stage and grade, smoking history, and BCG vaccination status.

^b^adjusted for Cis background, multifocality, and mBCG treatment.

^c^adjusted for age, gender, and smoking history.

^d^adjusted for age, gender, smoking history, and grade.

^e^adjusted for age and gender.

## References

[1] Babjuk M, Oosterlinck W, Sylvester R (2011). EAU guidelines on non-muscle-invasive urothelial carcinoma of the bladder, the 2011 update. *European Urology*.

[2] Han RF, Pan JG (2006). Can intravesical bacillus Calmette-Guérin reduce recurrence in patients with superficial bladder cancer? A meta-analysis of randomized trials. *Urology*.

[3] Shelley MD, Kynaston H, Court J (2001). A systematic review of intravesical bacillus Calmette-Guérin plus transurethral resection vs transurethral resection alone in Ta and T1 bladder cancer. *British Journal of Urology International*.

[4] Sylvester RJ, Van der Meijden APM, Lamm DL (2002). Intravesical bacillus Calmette-Guerin reduces the risk of progression in patients with superficial bladder cancer: a meta-analysis of the published results of randomized clinical trials. *Journal of Urology*.

[5] Brake M, Loertzer H, Horsch R, Keller H (2000). Recurrence and progression of stage T1, grade 3 transitional cell carcinoma of the bladder following intravesical immunotherapy with bacillus Calmette-Guerin. *Journal of Urology*.

[6] Brake M, Loertzer H, Horsch R, Keller H (2000). Long-term results of intravesical bacillus calmette-guérin therapy for stage T1 superficial bladder cancer. *Urology*.

[7] Fernandez-Gomez J, Solsona E, Unda M (2008). Prognostic factors in patients with non-muscle-invasive bladder cancer treated with bacillus Calmette-Guerin: multivariate analysis of data from four randomized CUETO trials. *European Urology*.

[8] Lamm DL (1985). Bacillus Calmette-Guerin immunotherapy for bladder cancer. *Journal of Urology*.

[9] Peyromaure M, Guerin F, Amsellem-Ouazana D, Saighi D, Debre B, Zerbib M (2003). Intravesical bacillus Calmette-Guerin therapy for stage T1 grade 3 transitional cell carcinoma of the bladder: recurrence, progression and survival in a study of 57 patients. *Journal of Urology*.

[10] Kresowik TP, Griffith TS (2009). Bacillus Calmette-Guerin immunotherapy for urothelial carcinoma of the bladder. *Immunotherapy*.

[11] Lamm DL, Van der Meijden APM, Morales A (1992). Incidence and treatment of complications of bacillus Calmette-Guerin intravesical therapy in superficial bladder cancer. *Journal of Urology*.

[12] Böhle A, Jocham D, Bock PR (2003). Intravesical bacillus Calmette-Guerin versus mitomycin C for superficial bladder cancer: a formal meta-analysis of comparative studies on recurrence and toxicity. *Journal of Urology*.

[13] Lamm DL, Blumenstein BA, Crissman JD (2000). Maintenance bacillus Calmette-Guerin immunotherapy for recurrent Ta, T1 and carcinoma in situ transitional cell carcinoma of the bladder: a randomized Southwest Oncology Group study. *Journal of Urology*.

[14] Van der Meijden APM, Sylvester RJ, Oosterlinck W, Hoeltl W, Bono AV (2003). Maintenance Bacillus Calmette-Guerin for Ta T1 bladder tumors is not associated with increased toxicity: results from a European organisation for research and treatment of cancer genito-urinary group phase III trial. *European Urology*.

[15] Schrohl AS, Holten-Andersen M, Sweep F (2003). Tumor markers: from laboratory to clinical utility. *Molecular & Cellular Proteomics*.

[16] Alexandroff AB, Nicholson S, Patel PM, Jackson AM (2010). Recent advances in bacillus Calmette-Guerin immunotherapy in bladder cancer. *Immunotherapy*.

[17] von Elm E, Altman DG, Egger M, Pocock SJ, Gøtzsche PC, Vandenbroucke JP (2008). The Strengthening the Reporting of Observational Studies in Epidemiology (STROBE) statement: guidelines for reporting observational studies. *Journal of Clinical Epidemiology*.

[18] Lacombe L, Dalbagni G, Zhang ZF (1996). Overexpression of p53 protein in a high-risk population of patients with superficial bladder cancer before and after Bacillus Calmette-Guerin therapy: correlation to clinical outcome. *Journal of Clinical Oncology*.

[19] Caliskan M, Türkeri LN (1997). Nuclear accumulation of mutant p53 protein: a possible predictor of failure of intravesical therapy in bladder cancer. *British Journal of Urology*.

[20] Okamura T, Akita H, Kawai N, Tozawa K, Yamada Y, Kohri K (1998). Immunohistochemical evaluation of p53, proliferating cell nuclear antigen (PCNA) and bcl-2 expression during bacillus calmette-guerin (BCG) intravesical instillation therapy for superficial bladder cancers. *Urological Research*.

[21] Lebret T, Becette V, Barbagelatta M (1998). Correlation between p53 over expression and response to bacillus calmette-guerin therapy in a high risk select population of patients with T1G3 bladder cancer. *Journal of Urology*.

[22] Zlotta AR, Noel JC, Fayt I (1999). Correlation and prognostic significance of p53, p21(WAF1/CIP1) and Ki-67 expression in patients with superficial bladder tumors treated with bacillus Calmette-Guerin intravesical therapy. *Journal of Urology*.

[23] Saint F, Le Frere Belda MA, Quintela R (2004). Pretreatment p53 nuclear overexpression as a prognostic marker in superficial bladder cancer treated with Bacillus Calmette-Guérin (BCG). *European Urology*.

[24] Peyromaure M, Weibing S, Sebe P (2002). Prognostic value of p53 overexpression in T1G3 bladder tumors treated with bacillus Calmette-Guérin therapy. *Urology*.

[25] Pages F, Flam TA, Vieillefond A (1998). p53 status does not predict initial clinical response to bacillus calmette-guerin intravesical therapy in T1 bladder tumors. *Journal of Urology*.

[26] Park J, Song C, Shin E, Hong JH, Kim CS, Ahn H (2011). Do molecular biomarkers have prognostic value in primary T1G3 bladder cancer treated with bacillus Calmette-Guerin intravesical therapy?. *Urologic Oncology*.

[27] Kyroudi-Voulgari A, Kouloukoussa M, Simigiatos C (2005). DNA ploidy and immunomarking of bladder urothelial tumors before and after intravesical bacillus Calmette-Guérin treatment. *Analytical and Quantitative Cytology and Histology*.

[28] Serdar A, Turhan C, Soner G (2005). The prognostic importance of e-cadherin and p53 gene expression in transitional bladder carcinoma patients. *International Urology and Nephrology*.

[29] Moyano Calvo JL, Blanco Palenciano E, Beato Moreno A (2006). Prognostic value of E-Cadherina, Beta Catenin, KI, Ki-67 antigen and p53 protein in the superficial bladder tumors. *Actas Urologicas Espanolas*.

[30] Esuvaranathan K, Chiong E, Thamboo TP (2007). Predictive value of p53 and pRb expression in superficial bladder cancer patients treated with BCG and interferon-alpha. *Cancer*.

[31] Palou J, Algaba F, Vera I, Rodriguez O, Villavicencio H, Sanchez-Carbayo M (2009). Protein expression patterns of ezrin are predictors of progression in T1G3 bladder tumours treated with nonmaintenance Bacillus Calmette-Guérin. *European Urology*.

[32] Cormio L, Tolve I, Annese P (2009). Altered p53 and pRb expression is predictive of response to BCG treatment in T1G3 bladder cancer. *Anticancer Research*.

[33] Moyano Calvo JL, De Miguel Rodríguez M, Poyato Galán JM (2001). Flow cytometry, DNA ploidy, Ki-67 label and overexpression of p53 protein in 121 T1 superficial bladder cancer. Retrospective study. 2nd Part: prognostic value and utility in the selection of the tretment with prophylactic BCG. *Actas Urologicas Espanolas*.

[34] Lopez-Beltran A, Luque RJ, Alvarez-Kindelan J (2004). Prognostic factors in stage T1 grade 3 bladder cancer survival: the role of G1-S modulators (p53, p21Waf1, p27kip1, Cyclin D1, and Cyclin D3) and proliferation index (ki67-MIB1). *European Urology*.

[35] Lee E, Park I, Lee C (1997). Prognostic markers of intravesical bacillus Calmette-Guerin therapy for multiple, high-grade, stage T1 bladder cancers. *International Journal of Urology*.

[36] Pfister C, Flaman JM, Dunet F, Grise P, Frebourg T (1999). p53 mutations in bladder tumors inactivate the transactivation of the p21 and Bax genes, and have a predictive value for the clinical outcome after bacillus Calmette-Guerin therapy. *Journal of Urology*.

[37] Lebret T, Becette V, Hervé JM (2000). Prognostic value of MIB-1 antibody labeling index to predict response to bacillus Calmette-Guerin therapy in a high-risk selected population of patients with stage T1 grade G3 bladder cancer. *European Urology*.

[38] Blanchet P, Droupy S, Eschwege P (2001). Prospective evaluation of Ki-67 labeling in predicting the recurrence and progression of superficial bladder transitional cell carcinoma. *European Urology*.

[39] Cormio L, Tolve I, Annese P (2010). Retinoblastoma protein expression predicts response to bacillus Calmette-Guérin immunotherapy in patients with T1G3 bladder cancer. *Urologic Oncology*.

[40] Mantovani A, Germano G, Marchesi F, Locatelli M, Biswas SK (2011). Cancer-promoting tumor-associated macrophages: new vistas and open questions. *European Journal of Immunology*.

[41] Ayari C, LaRue H, Hovington H (2009). Bladder tumor infiltrating mature dendritic cells and macrophages as predictors of response to Bacillus Calmette-Guérin immunotherapy. *European Urology*.

[42] Takayama H, Nishimura K, Tsujimura A (2009). Increased infiltration of tumor associated macrophages is associated with poor prognosis of bladder carcinoma in situ after intravesical bacillus Calmette-Guerin instillation. *Journal of Urology*.

[43] Janane A, Hajji F, Ismail TO (2011). Evaluation of HER2 protein overexpression in non-muscle invasive bladder cancer with emphasis on tumour grade and recurrence. *Actas Urologicas Espanolas*.

[44] Lopez-Beltran A, Ordóñez JL, Otero AP (2010). Cyclin D3 gene amplification in bladder carcinoma in situ. *Virchows Archiv*.

[45] Kim SIL, Kwon SM, Kim YS, Hong SJ (2002). Association of cyclooxygenase-2 expression with prognosis of stage T1 grade 3 bladder cancer. *Urology*.

[46] Yutkin V, Pode D, Pikarsky E, Mandelboim O (2007). The expression level of ligands for natural killer cell receptors predicts response to bacillus Calmette-Guerin therapy: a pilot study. *Journal of Urology*.

[47] Lebret T, Watson RWG, Molinié V (2007). HSP90 expression: a new predictive factor for BCG response in stage Ta-T1 grade 3 bladder tumours. *European Urology*.

[48] Kitamura H, Torigoe T, Honma I (2006). Effect of human leukocyte antigen class I expression of tumor cells on outcome of intravesical instillation of Bacillus Calmette-Guerin immunotherapy for bladder cancer. *Clinical Cancer Research*.

[49] Gazzaniga P, Gradilone A, De Berardinis E (2009). A chemosensitivity test to individualize intravesical treatment for non-muscle-invasive bladder cancer. *British Journal of Urology International*.

[50] Videira PA, Calais FM, Correia M (2009). Efficacy of bacille Calmette-Guerin immunotherapy predicted by expression of antigen-presenting molecules and chemokines. *Urology*.

[51] Kim YJ, Ha YS, Kim SK (2010). Gene signatures for the prediction of response to Bacillus Calmette-Guérin immunotherapy in primary pT1 bladder cancers. *Clinical Cancer Research*.

[52] Alvarez-Múgica M, Cebrian V, Fernández-Gómez JM, Fresno F, Escaf S, Sánchez-Carbayo M (2010). Myopodin methylation is associated with clinical outcome in patients with T1G3 bladder cancer. *Journal of Urology*.

[53] Agundez M, Grau L, Palou J, Algaba F, Villavicencio H, Sanchez-Carbayo M (2011). Evaluation of the methylation status of tumour suppressor genes for predicting bacillus Calmette-Guérin response in patients with T1G3 high-risk bladder tumours. *European Urology*.

[54] Sagnak L, Ersoy H, Ozok U (2009). Predictive value of urinary interleukin-8 cutoff point for recurrences after transurethral resection plus induction bacillus calmette-guérin treatment in non-muscle-invasive bladder tumors. *Clinical Genitourinary Cancer*.

[55] Shintani Y, Sawada Y, Inagaki T, Kohjimoto Y, Uekado Y, Shinka T (2007). Intravesical instillation therapy with bacillus Calmette-Guérin for superficial bladder cancer: study of the mechanism of bacillus Calmette-Guérin immunotherapy. *International Journal of Urology*.

[56] Watanabe E, Matsuyama H, Matsuda K (2003). Urinary interleukin-2 may predict clinical outcome of intravesical bacillus Calmette-Guérin immunotherapy for carcinoma in situ of the bladder. *Cancer Immunology, Immunotherapy*.

[57] Kumar A, Dubey D, Bansal P, Mandhani A, Naik S (2002). Urinary interleukin-8 predicts the response of standard and low dose intravesical bacillus calmette-guerin (modified Danish 1331 strain) for superficial bladder cancer. *Journal of Urology*.

[58] Sánchez-Carbayo M, Urrutia M, Romani R, Herrero M, Gonzalez De Buitrago JM, Navajo JA (2001). Serial urinary IL-2, IL-6, IL-8, TNF*α*, UBC, CYFRA 21-1 and NMP22 during follow-up of patients with bladder cancer receiving intravesical BCG. *Anticancer Research*.

[59] Thalmann GN, Sermier A, Rentsch C, Möhrle K, Cecchini MG, Studer UE (2000). Urinary interleukin-8 and 18 predict the response of superficial bladder cancer to intravesical therapy with bacillus Calmette-Guerin. *Journal of Urology*.

[60] Jackson AM, Ivshina AV, Senko O (1998). Prognosis of intravesical bacillus calmette-guerin therapy for superficial bladder cancer by immunological urinary measurements: statistically weighted syndromes analysis. *Journal of Urology*.

[61] Thalmann GN, Dewald B, Baggiolini M, Studer UE (1997). Interleukin-8 expression in the urine after bacillus Calmette-Guerin therapy: a potential prognostic factor of tumor recurrence and progression. *Journal of Urology*.

[62] Rabinowitz R, Smith DS, Tiemann DD, Hudson MA (1997). Urinary interleukin-8/creatinine level as a predictor of response to intravesical bacillus Calmette-Guerin therapy in bladder tumor patients. *Journal of Urology*.

[63] Saint F, Kurth N, Maille P (2003). Urinary IL-2 assay for monitoring intravesical bacillus Calmette-Guérin response of superficial bladder cancer during induction course and maintenance therapy. *International Journal of Cancer*.

[64] Saint F, Patard JJ, Maille P (2002). Prognostic value of a T helper 1 urinary cytokine response after intravesical bacillus Calmette-Guerin treatment for superficial bladder cancer. *Journal of Urology*.

[65] Saint F, Patard JJ, Maille P (2001). T helper 1/2 lymphocyte urinary cytokine profiles in responding and nonresponding patients after 1 and 2 courses of bacillus calmette-guerin for superficial bladder cancer. *Journal of Urology*.

[66] De Reijke TM, De Boer EC, Kurth KH, Schamhart DHJ (1996). Urinary cytokines during intravesical bacillus Calmette-Guerin therapy for superficial bladder cancer: processing, stability and prognostic value. *Journal of Urology*.

[67] Eto M, Koga H, Noma H, Yamaguchi A, Yoshikai Y, Naito S (2005). Importance of urinary interleukin-18 in intravesical immunotherapy with bacillus Calmette-Guérin for superficial bladder tumors. *Urologia Internationalis*.

[68] Hausladen DA, Wheeler MA, Altieri DC, Colberg JW, Weiss RM (2003). Effect of intravesical treatment of transitional cell carcinoma with bacillus Calmette-Guerin and mitomycin C on urinary survivin levels and outcome. *Journal of Urology*.

[69] Danişman A, Bulut K, Kukul E, Özen I, Sevük M (2000). Urinary fibronectin levels in patients treated with intravesical bacillus Calmette-Guerin for superficial bladder cancer. *Urologia Internationalis*.

[70] Kaempfer R, Gerez L, Farbstein H (1996). Prediction of response to treatment in superficial bladder carcinoma through pattern of interleukin-2 gene expression. *Journal of Clinical Oncology*.

[71] Magno C, Melloni D, Galì A (2002). The anti-tumor activity of bacillus Calmette-Guerin in bladder cancer is associated with an increase in the circulating level of interleukin-2. *Immunology Letters*.

[72] Bilen CY, Inci K, Erkan I, Özen H (2003). The predictive value of purified protein derivative results on complications and prognosis in patients with bladder cancer treated with bacillus Calmette-Guerin. *Journal of Urology*.

[73] Chiong E, Kesavan A, Mahendran R (2011). NRAMP1 and hGPX1 gene polymorphism and response to bacillus Calmette-Guérin therapy for bladder cancer. *European Urology*.

[74] Decobert M, LaRue H, Bergeron A (2006). Polymorphisms of the human NRAMP1 gene are associated with response to bacillus Calmette-Guerin immunotherapy for superficial bladder cancer. *Journal of Urology*.

[75] Gu J, Zhao H, Dinney CP (2005). Nucleotide excision repair gene polymorphisms and recurrence after treatment for superficial bladder cancer. *Clinical Cancer Research*.

[76] Gangwar R, Mandhani A, Mittal RD (2010). XPC gene variants: a risk factor for recurrence of urothelial bladder carcinoma in patients on BCG immunotherapy. *Journal of Cancer Research and Clinical Oncology*.

[77] Gangawar R, Ahirwar D, Mandhani A, Mittal RD (2010). Impact of nucleotide excision repair ERCC2 and base excision repair APEX1 genes polymorphism and its association with recurrence after adjuvant BCG immunotherapy in bladder cancer patients of North India. *Medical Oncology*.

[78] Mittal RD, Singh R, Manchanda PK (2008). XRCC1 codon 399 mutant allele: a risk factor for recurrence of urothelial bladder carcinoma in patients on BCG immunotherapy. *Cancer Biology & Therapy*.

[79] Mittal RD, Gangwar R, Mandal RK, Srivastava P, Ahirwar DK (2011). Gene variants of XRCC4 and XRCC3 and their association with risk for urothelial bladder cancer. *Molecular Biology Reports*.

[80] Ahirwar DK, Agrahari A, Mandhani A, Mittal RD (2009). Cytokine gene polymorphisms are associated with risk of urinary bladder cancer and recurrence after BCG immunotherapy. *Biomarkers*.

[81] Ahirwar DK, Mandhani A, Mittal RD (2010). IL-8 -251 T > A Polymorphism associated with bladder cancer susceptibility and outcome after BCG immunotherapy in a northern Indian cohort. *Archives of Medical Research*.

[82] Cai T, Nesi G, Dal Canto M (2010). Loss of heterozygosis on IFN-alpha locus is a prognostic indicator of bacillus Calmette-Guerin response for nonmuscle invasive bladder cancer. *Journal of Urology*.

[83] Gangwar R, Mandhani A, Mittal RD (2011). Functional polymorphisms of cyclooxygenase-2 (COX-2) gene and risk for urinary bladder cancer in North India. *Surgery*.

[84] Ahirwar D, Kesarwani P, Manchanda PK, Mandhani A, Mittal RD (2008). Anti- and proinflammatory cytokine gene polymorphism and genetic predisposition: association with smoking, tumor stage and grade, and bacillus Calmette-Guérin immunotherapy in bladder cancer. *Cancer Genetics and Cytogenetics*.

[85] Ahirwar DK, Mandhani A, Dharaskar A, Kesarwani P, Mittal RD (2009). Association of tumour necrosis factor-*α* gene (T-1031C, C-863A, and C-857T) polymorphisms with bladder cancer susceptibility and outcome after bacille Calmette-Guérin immunotherapy. *British Journal of Urology International*.

[86] Leibovici D, Grossman HB, Dinney CP (2005). Polymorphisms in inflammation genes and bladder cancer: from initiation to recurrence, progression, and survival. *Journal of Clinical Oncology*.

[87] Baştürk B, Yavaşçaoğlu I, Vuruşkan H, Göral G, Oktay B, Oral HB (2005). Cytokine gene polymorphisms as potential risk and protective factors in renal cell carcinoma. *Cytokine*.

[88] Gangwar R, Mandhani A, Mittal RD (2009). Caspase 9 and Caspase 8 gene polymorphisms and susceptibility to bladder cancer in North Indian population. *Annals of Surgical Oncology*.

[89] Gangwar R, Mittal RD (2010). Association of selected variants in genes involved in cell cycle and apoptosis with bladder cancer risk in north Indian population. *DNA and Cell Biology*.

[90] Srivastava P, Gangwar R, Kapoor R, Mittal RD (2010). Bladder cancer risk associated with genotypic polymorphism of the matrix metalloproteinase-1 and 7 in North Indian population. *Disease Markers*.

[91] Srivastava P, Kapoor R, Mittal RD (2011). Association of single nucleotide polymorphisms in promoter of matrix metalloproteinase-2, 8 genes with bladder cancer risk in Northern India. *Urologic Oncology*.

[92] Srivastava P, Mandhani A, Kapoor R, Mittal RD (2010). Role of MMP-3 and MMP-9 and their haplotypes in risk of bladder cancer in North Indian cohort. *Annals of Surgical Oncology*.

[93] Chen M, Hildebrandt MAT, Clague J (2010). Genetic variations in the sonic hedgehog pathway affect clinical outcomes in non-muscle-invasive bladder cancer. *Cancer Prevention Research*.

[94] Zuiverloon TCM, Nieuweboer AJM, Vékony H, Kirkels WJ, Bangma CH, Zwarthoff EC (2012). Markers predicting response to bacillus Calmette-Guérin immunotherapy in high-risk bladder cancer patients: a systematic review. *European Urology*.

[95] Morgan BE, Salup R, Morgan MB (2002). Differential C-erbB-2 and VEGF expression following BCG immunotherapy in superficial papillary transitional cell carcinoma of the bladder. *Urologic Oncology*.

[96] Zachos I, Konstantinopoulos PA, Vandoros GP (2009). Predictive value of telomerase reverse transcriptase expression in patients with high risk superficial bladder cancer treated with adjuvant BCG immunotherapy. *Journal of Cancer Research and Clinical Oncology*.

[97] Inman BA, Sebo TJ, Frigola X (2007). PD-L1 (B7-H1) expression by urothelial carcinoma of the bladder and BCG-induced granulomata: associations with localized stage progression. *Cancer*.

[98] Honda SI, Sakamoto Y, Fujime M, Kitagawa R (1997). Immunohistochemical study of tumor-infiltrating lymphocytes before and after intravesical bacillus Calmette-Guerin treatment for superficial bladder cancer. *International Journal of Urology*.

[99] Ardelt PU, Kneitz B, Adam P (2010). Reactive antibodies against bacillus Calmette-Guerin heat-shock protein-65 potentially predict the outcome of immunotherapy for high-grade transitional cell carcinoma of the bladder. *Cancer*.

[100] Whitson J, Berry A, Carroll P, Konety B (2009). A multicolour fluorescence in situ hybridization test predicts recurrence in patients with high-risk superficial bladder tumours undergoing intravesical therapy. *British Journal of Urology International*.

[101] Savic S, Zlobec I, Thalmann GN (2009). The prognostic value of cytology and fluorescence in situ hybridization in the follow-up of nonmuscle-invasive bladder cancer after intravesical Bacillus Calmette-Guérin therapy. *International Journal of Cancer*.

[102] Mengual L, Marín-Aguilera M, Ribal MJ (2007). Clinical utility of fluorescent in situ hybridization for the surveillance of bladder cancer patients treated with bacillus Calmette-Guerin therapy. *European Urology*.

[103] Kipp BR, Karnes RJ, Brankley SM (2005). Monitoring intravesical therapy for superficial bladder cancer using fluorescence in situ hybridization. *Journal of Urology*.

[104] Saint F, Patard JJ, Irani J (2001). Leukocyturia as a predictor of tolerance and efficacy of intravesical BCG maintenance therapy for superficial bladder cancer. *Urology*.

